# From theory to practice: examining the influence of physically active learning on curriculum design and pedagogical planning in initial teacher education

**DOI:** 10.3389/fspor.2025.1719341

**Published:** 2025-11-26

**Authors:** Natalie Lander, Kira Patterson, Samuel K. Lai, Nicole Martin-Alcaide, Jess Orr, Ned Weatherell, Kim Beasy, Jo Salmon

**Affiliations:** 1 School of Exercise and Nutrition Sciences, Institute for Physical Activity and Nutrition (IPAN), Deakin University, Geelong, VIC, Australia; 2 School of Education, University of Tasmania, Launceston, TAS, Australia

**Keywords:** physically active learning, initial teacher education, pre-service teachers, lesson planning, active pedagogies

## Abstract

**Introduction:**

Teacher effectiveness is a critical factor influencing student outcomes, with physically active learning (PAL) emerging as an evidence-based approach that enhances academic achievement and health. Embedding PAL within Initial Teacher Education (ITE) may support pre-service teachers to develop pedagogical competence and confidence. TransformUs Higher Ed is a research-informed intervention designed to integrate PAL within ITE, aligned with the Australian Professional Standards for Teachers—Graduate Level. While prior research shows improvements in pre-service teacher confidence and willingness to use PAL, limited evidence exists on how these strategies are embedded in pre-service teachers' lesson planning. This study explored how components, philosophies, and strategies from TransformUs Higher Ed were incorporated into the lesson planning of first-year Bachelor of Education pre-service teachers at an Australian university.

**Methods:**

A mixed-methods study was conducted with first-year Bachelor of Education pre-service teachers (*N* = 141). Participants received the TransformUs Higher Ed program as part of a core Curriculum and Pedagogy unit. Quantitative data were extracted from lesson sequence assessments, capturing inclusion, frequency, type, and purpose of PAL strategies and their alignment with professional standards. Descriptive statistics were generated using Stata SE 18. Qualitative data were drawn from students' video reflections and analysed inductively in NVivo 14.

**Results:**

Of the 141 participants, 89% incorporated at least one PAL strategy into lesson planning, yielding 447 active strategies (*M* = 3.17 per student). Active breaks, particularly transition- and structure-based breaks, were more common than active lessons, with experiential learning the most frequent lesson type. Strategies were typically applied during the lesson body and aligned with Standard 3: Plan for and implement effective teaching and learning. Thematic analysis identified eight pedagogical themes, including Learning Through Doing, Constructivist Learning, and Collaborative Learning. Findings demonstrate the utility of TransformUs Higher Ed in promoting PAL adoption within lesson planning, supporting pedagogical competence and alignment with evidence-based teaching standards.

**Conclusion:**

Embedding PAL-focused interventions within ITE programs can foster pedagogical competence, with lesson planning serving as a crucial step in developing effective, evidence-based teaching practices among future educators.

## 1 Introduction

Teacher effectiveness is a key determinant of student success, with a substantial body of research demonstrating a strong relationship between teacher quality and student achievement (1–3). Of all in-school factors, the quality of teaching is consistently identified as having the greatest impact on student learning outcomes, surpassing the influence of curriculum, school leadership, or available resources (2). Effective teaching is commonly linked to a combination of teacher experience, content knowledge, and pedagogical practices, each of which plays a critical role in shaping student engagement, understanding, and academic performance (3–7). High-quality teaching involves not only mastery of subject matter but also the ability to translate that knowledge into responsive and inclusive instructional strategies that meet diverse learner needs (2, 7). Effective teaching is central to achieving equitable and meaningful learning, positioning instructional effectiveness and pedagogical practice as the most critical mechanisms for improving educational outcomes (7).

Among the pedagogical approaches shown to enhance educational outcomes is Physically Active learning (PAL). PAL involves embedding physical activity into the delivery of academic content to improve both educational and health outcomes (8). PAL can be implemented through multiple modalities, as part of the structure of a lesson, using active breaks to transition between learning activities or re-energize students following extended periods of sedentary instruction (9); through the physical environment, such as the use of standing desks, floor markings, or wall signage to promote movement; and as a pedagogical approach, drawing on experiential or embodied learning principles that enable students to learn by doing (8, 10–15). These strategies have been found to improve both academic (11, 12, 16–20) and health outcomes (14, 21, 22) in students. Emerging evidence further highlights the positive effects of active teaching on key developmental domains such as concentration, classroom behaviour, cognitive function, emotional regulation, impulse control, attitude and intrinsic motivation (14, 17, 23, 24).

In addition, PAL facilitates several evidence-based pedagogical strategies, including collaborative opportunities for learning, differentiation, multiple exposures of content, and the use of metacognitive strategies (9). Meta-analyses indicate that these pedagogical strategies positively influence learning and are consistently associated with improved student outcomes across diverse educational settings (25, 26). Collectively, these findings underscore the value of integrating PAL within everyday classroom practice to support student learning outcomes, aligning with the World Health Organization's *Promoting Physical Activity Through Schools* toolkit, which advocates for the inclusion of PAL strategies alongside high-quality physical education (PE) to advance global priorities in children and adolescent's health and education (27).

Given the well-documented links between PAL and improved educational and health outcomes for students, research in this area has grown considerably in recent years (13, 18, 28). One notable example is the *TransformUs* intervention, a comprehensive school-based program that integrates behavioural, environmental, and pedagogical strategies to reduce sedentary behaviour, increase physical activity, and enhance learning and health outcomes (29–31). While this program has demonstrated success in supporting in-service teachers to adopt more active pedagogies, ongoing professional development for practicing teachers is often constrained by competing demands, time limitations, and entrenched instructional habits (32–34). These barriers highlight the need for more sustainable, upstream approaches. Embedding PAL within initial teacher education (ITE) programs offers a promising alternative, providing pre-service teachers with early exposure to active instructional strategies. This not only promotes the development of pedagogical competence but also supports long-term teaching efficacy by fostering innovative practices from the outset of their careers (35, 36).

ITE programs are fundamental in preparing future educators with the necessary pedagogical knowledge, skills, and confidence to foster effective learning (37, 38). ITE programs are designed to develop the personal and professional capacities of future teachers, including self-efficacy, content knowledge, and pedagogical content knowledge (37, 38). Incorporating interactive and transformative pedagogies within ITE allows pre-service teachers to experiment with and apply their learning, fostering the development of new knowledge and refined teaching practices (39), and is essential for driving long-term, sustainable change in schools (40). Traditionally, PE teachers have been primarily responsible for students' physical activity. However, generalist teachers spend far more time with students than PE specialists, making it essential to develop their competencies in PAL during ITE. This not only complements the work of PE teachers but also equips generalist teachers to implement evidence-based strategies that enhance student engagement and academic outcomes.

A crucial component of ITE is the development of lesson planning skills, which enable future teachers to design clear, engaging lessons that align with educational objectives (41–43). Effective lesson planning is integral to creating meaningful learning experiences, managing classroom dynamics, and assessing student progress. Furthermore, it serves as a foundation for reflective teaching practices and ongoing professional development (41). In ITE, the cultivation of lesson planning and sequencing abilities is essential for preparing teachers who can effectively translate the curriculum into structured, responsive learning experiences. By equipping pre-service teachers with the skills to make informed decisions about content selection, instructional strategies and assessment, lesson planning ensures that diverse student needs are met and student achievement is promoted (44). Furthermore, the sequencing of lessons ensures a progressive layering of learning concepts, scaffolding each lesson to reinforce and extend student understanding over time. These competencies also enhance pedagogical content knowledge, allowing teachers to align their instruction with curriculum standards while adapting to the evolving dynamics of the classroom (45, 46). For pre-service teachers, mastering the process of lesson planning and sequencing is vital for fostering pedagogical competence. Additionally, the act of planning and sequencing encourages reflective practice, a critical aspect of effective teaching, as it prompts pre-service teachers to consider the rationale behind their instructional choices and their impact on student learning. Ultimately, the development of these skills contributes to teacher confidence, classroom preparedness, and greater student outcomes (45).

*TransformUs Higher Ed* is a research-informed intervention adapted from the successful *TransformUs* program, specifically designed for integration within ITE. It adopts a multi-level approach, addressing behavioural, pedagogical, and environmental factors, to prepare future teachers with practical, innovative strategies for increasing learning engagement and outcomes through PAL (36, 47–49). The intervention is closely aligned with the Australian Professional Standards for Teachers at the Graduate level (50), ensuring that it supports the development of essential teaching competencies (50). Findings from pilot feasibility, effectiveness and implementation studies (36, 47–49) have demonstrated improvements in pre-service teachers' confidence, competence, and willingness to implement PAL strategies. Qualitative data further revealed that the program helped bridge the gap between university-based learning and the practical demands of school teaching (48). These studies also identified key areas for refinement and future research, including the importance of incorporating *TransformUs Higher Ed* concepts and strategies into lesson planning and sequencing to foster pedagogical competence.

The present study aimed to address this gap by exploring how the components, philosophies, and strategies from the *TransformUs Higher Ed* program are incorporated into the lesson planning of first-year Bachelor of Education students at an Australian university. Specifically, the research aimed to: (1) determine the number of pre-service teachers who included at least one active strategy from *TransformUs Higher Ed* in their three-lesson sequence; (2) assess the prevalence and frequency of the PAL strategies; (3) analyse the types of strategies included, namely, active environments, active lessons, and active breaks, and identify the most commonly used approaches; and (4) explore the purpose of the PAL strategies within the lessons, by investigating where in the lesson (introduction, body, conclusion), as well as how and why they were used (i.e., alignment with evidence-based pedagogical/teaching strategies and evidence of the Australian Professional Standards for Teachers –Graduate Level).

## 2 Methods

### 2.1 Study design

This study employed a mixed-methods design, underpinned by a pragmatic epistemological stance (51) that values multiple forms of data to address complex educational questions. Drawing on elements of constructivist and practice theory, the research recognises that pre-service teachers develop pedagogical knowledge through authentic engagement with curriculum planning tasks and reflection on teaching practices (52–54).

In alignment with the study's aims, four research questions guided the investigation:

1. To what extent did pre-service teachers include at least one active strategy from TransformUs Higher Ed in their three-lesson sequence?
2. How frequently were PAL strategies incorporated across lessons?
3. What types of PAL strategies (active environments, active lessons, or active breaks) were used most?
4. For what purposes and at what points in the lesson (introduction, body, conclusion) were PAL strategies implemented, and how did these align with evidence-based pedagogy and the Australian Professional Standards for Teachers (Graduate Level)?

To address the aims and research questions, quantitative data were extracted from the three-lesson sequence assessment task submitted via PebblePad and analysed descriptively to quantify the inclusion, distribution and prevalence of *TransformUs* strategies (Aims 1–4). To further address Aim 4, qualitative data were drawn from students' accompanying reflective video submissions, in which they articulated the rationale behind their pedagogical choices. A thematic analysis of these videos was conducted to investigate how and why active strategies were integrated into lesson design, and how pre-service teachers linked these choices to broader educational models, frameworks, and standards.

### 2.2 Context

The TransformUs Higher Ed intervention was implemented in the *Curriculum and Pedagogy* unit, a core first-year subject within the Bachelor of Education program at one Australian university during Semester 2, 2024. The unit provides pre-service teachers with a foundational understanding of curriculum frameworks and pedagogical approaches that underpin effective teaching and learning. It also encourages critical reflection on how curriculum content and delivery are influenced by broader social, political, and economic forces (55). Students engage with theoretical and practical aspects of curriculum design and pedagogy, emphasizing innovation, professionalism, ethics, and equity in teaching practice.

The unit's major assessment, *Understanding Curriculum, Pedagogy, and Planning*, required students to design a three-lesson sequence aligned with the Australian Curriculum and submit it as a digital portfolio via PebblePad. Using a structured MyLO template (the university's online learning platform), students demonstrated their ability to integrate curriculum knowledge, pedagogical theory, and classroom implementation strategies. To complement the written component, students produced short video reflections responding to audience-specific prompts, such as explaining their pedagogical choices to a fellow teacher. This multimodal assessment encouraged critical reflection, professional communication, and justification of instructional decisions from multiple educational perspectives.

### 2.3 Participants

All pre-service teachers enrolled in the first-year *Curriculum and Pedagogy* unit of the Bachelor of Education program (*N* = 156) were invited to participate in the study. As part of the unit, all pre-service teachers received the *TransformUs Higher Education* content integrated throughout the semester. The primary data source for the study was the major assessment task completed within this unit. An opt-out consent process was used, whereby students were informed that their assessment data could be included in the study unless they chose to withdraw. A total of 11 students opted out, resulting in a final sample of 145 pre-service teachers whose data were included in the analysis. The study was approved by Deakin University (HAE-20-170) and University of Tasmania (30827) ethics committees.

### 2.4 Intervention phases

The intervention was implemented in three structured phases, each designed to support the integration of the *TransformUs Higher Ed* program into the Curriculum and Pedagogy unit. The focus was on embedding PAL strategies (e.g., active break, active lessons, active environments) aligned with unit learning outcomes, evidence-based teaching frameworks, models and practices, and the Australian Professional Standards for Teachers—Graduate Level.

#### 2.4.1 Phase 1: program alignment and staff development

This phase involved collaborative planning between the research team and senior academic staff, including the Course Director and Unit Chair. The objective was to introduce the *TransformUs Higher Ed* program and align its philosophies, components, and strategies with the goals of ITE and the specific learning outcomes of the Curriculum and Pedagogy unit. This initial collaboration enabled the identification of relevant Bachelor of Education staff, fostering a meaningful integration of *TransformUs Higher Ed* principles to enhance the unit curriculum.

To operationalise this alignment, the lead researcher and Unit Chair subsequently co-facilitated a 2-h professional development workshop for lecturers and sessional staff. The workshop provided an overview of *TransformUs Higher Ed* and its relevance to unit content, with explicit alignment to evidence-based teaching methods, the Australian Professional Standards for Teachers—Graduate Level, and relevant pedagogical and instructional models and frameworks (e.g., *Learners First*, *Gradual Release of Responsibility Model*, *5E Instructional Model*, and *Bloom's Taxonomy*) (50, 56). The workshop included an interactive component, allowing staff to experience PAL strategies firsthand and offering dedicated planning time to embed these approaches into their teaching practice.

#### 2.4.2 Phase 2: pre-service teacher engagement

##### 2.4.2.1 Guest lecture

In Week 6 of the semester, the lead researcher delivered a guest lecture to introduce *TransformUs Higher Ed* to pre-service teachers enrolled in the Curriculum and Pedagogy unit. The lecture was recorded and housed on the unit's online platform. The session outlined the program's rationale, key components, alignment with AITSL's Graduate Standards (50), and relevance to both unit and broader ITE objectives. Pre-service teachers were provided guided access to the *TransformUs* website (https://transformus.com.au/), which offers an extensive bank of online resources, including over 150 two-minute active breaks and more than 100 active academic lessons across Foundation to Year 10 levels in key curriculum areas such as mathematics, science, English, geography, and history. All *TransformUs* resources are linked to the Australian Curriculum and accompanied by online professional learning materials, instructional models, pedagogical guidance, and practical implementation tools.

During the workshop, pre-service teachers explored active teaching resources and engaged in scenario-based practice, where they planned, delivered, and reflected on sample lessons incorporating active breaks and active academic content.

##### 2.4.2.2 Targeted active teaching and learning lectures

In Weeks 8 and 9, the research team in collaboration with the unit teaching staff delivered targeted lectures and tutorials exploring how *TransformUs Higher Ed* can enhance the application of pedagogical frameworks (e.g., *Learners First*), instructional models (e.g., *Gradual Release of Responsibility*, 5*E's Model*), and educational frameworks (e.g., *Bloom's Taxonomy*). These sessions included interactive tasks, case studies, and scenario-based learning activities. Pre-service teachers engaged with the *TransformUs* resources to complete targeted tasks such as designing lesson scaffolds aligned to Bloom's taxonomy using active teaching strategies and selecting and analysing one active break and one active lesson to demonstrate how they could be used to differentiate instruction by content, process, or product.

#### 2.4.3 Phase 3: modelling and integration of active teaching strategies

In the final phase, lecturers were encouraged to model PAL strategies in their own seminars and lectures across the semester, embed *TransformUs Higher Ed* (9, 36, 47, 49) principles into their instructional practices and provide ongoing references to pedagogical, instructional, and educational frameworks covered in the unit.

The final unit assessment required pre-service teachers to design a sequence of three lesson plans for a chosen school year level and subject, accompanied by a video explaining their pedagogical approach. The inclusion of PAL strategies was not a formal requirement of the assessment and was not assessed in the rubric of the unit.

### 2.5 Data collection

The research team, comprising three experienced teachers, researchers in physical activity and teacher education academics, developed a structured template to guide the data extraction process from the lesson plans and accompanying video reflections. The template was designed to capture key indicators of PAL, effective lesson planning, evidence-based instructional practices, and alignment with the Australian Professional Standards for Teachers—Graduate Level (50).

Table 1 provides an overview of each component of the template, including definitions, relevant indicators, and their alignment to the Graduate Level Standards and the broader aims of the research. This template served as an analytical framework for identifying if, where, how, and why pre-service teachers incorporated active teaching strategies into their lessons.

**Table 1:** Template for data extraction

Domain	Item	Elaboration	Evidence	Link to study aim
Physically active instructional approaches/physically active learning (PAL)	Creating an active classroom environment.	The use of signage, equipment, facilities, resources, classroom layout/desk configuration and policy, to support or promote meaningful physical activity in the classroom	(10)	1, 2, 3
	Integrating active breaks	The use of short active breaks to interrupt prolonged sitting and complement lesson content. Active breaks can be used for physical and visual reinforcement, to introduce or summarise lesson content, to structure the lesson, to transition the lesson, to proactively manage the class, and to create a positive classroom environment.	(9, 10)	1, 2, 3
		Types of Active Breaks:		
		Structure:		
		To use movement as part of the structure of the lesson		
		Transition:		
		To allow intentional and task-oriented movement as students transition between learning tasks or lesson phases		
		Manage:		
		To proactively and positively manage the classroom		
		Energise:		
		To break up long periods of sedentary class time and reenergize students with short bouts of physical activity		
		Learn:		
		To introduce, reinforce, consolidate, or demonstrate learning in a physically active way		
	Delivering active academic lessons	Active lessons utilize incidental or structural activity, or embodied or experiential learning, to change the delivery of a traditional seated class lesson to one where the body or the movement becomes a vehicle for learning.	(9, 10, 36, 47, 49)	1, 2, 3
		Types of Active Lessons:		
		Embodied learning refers to an approach in which physical movement and bodily experiences are integrated into the learning process. This approach emphasizes the connection between the body and mind, where students engage in activities that require physical involvement, such as gesture, movement, or role-playing. The theory suggests that the body’s involvement in learning can enhance cognitive processes, memory retention, and understanding by creating deeper, more meaningful connections to the material being studied.		
		Experiential learning is a process where learners gain knowledge and skills through direct hands-on experience, rather than through traditional academic instruction or passive learning methods. It involves actively engaging with real-world situations (e.g., experiments) or problem-solving tasks, reflecting on those experiences, and applying insights gained to future scenarios.		
		Structural or incidental movement refers to the intentional incorporation of physical activity or movement into a lesson, which is either a planned part of the lesson structure (e.g., station work) or occurs naturally during student interactions (e.g., standing and collaborating in small groups). This can include activities such as moving between stations, engaging in group discussions while standing, or participating in interactive tasks that require physical engagement.		
Lesson phase	Introduction	To capture students’ attention, activate prior knowledge, and set the context for the lesson. It helps students understand the learning objectives and prepares them for new information.	(2, 50, 66, 67)	4
	Body	The body of the lesson involves the core content, where new concepts, skills, or knowledge are taught through various activities, explanations, and discussions.	(2, 50, 67, 69)	4
	Conclusion	To summarize key points, reinforce learning, and assess understanding, allowing for reflection or clarification. A clear conclusion consolidates learning, helping students retain information and transfer it to future contexts, while also providing an opportunity for feedback.	(2, 50, 67, 69)	4
Evidence-based teaching practices	Goal Setting/Setting objectives	Goal setting is used to define clear, specific objectives that guide instructional strategies and student learning outcomes. It is a systematic process that enables teachers to articulate what they want students to achieve during a lesson or unit, ensuring that the teaching aligns with curriculum standards and student needs.	(70, 71)	4
	Structuring Lessons	Structuring lessons refers to the deliberate organization and sequencing of instructional activities to promote optimal student engagement and learning.	(50)	4
	Collaborative Learning	Providing opportunities for students to work together in small groups to achieve a common learning goal. This method emphasizes peer interaction, active participation, and the exchange of ideas, enabling students to engage deeply with the material through discussion, problem-solving, and shared responsibilities.	(72)	4
	Metacognitive Strategies	Metacognitive strategies, refer to the techniques used to help students become aware of and regulate their own thinking and learning processes, improve their ability to plan, monitor, and evaluate their understanding.	(68)	4
	Explicit Teaching	Explicit teaching involves clear instructions, modelling, and scaffolded learning experiences to ensure that students understand and apply concepts effectively.	(2, 66)	4
	Multiple exposures	Using repeated, varied exposures to content to help students consolidate learning over time, ensuring better retention and understanding.	(50)	4
	Differentiation	Differentiated teaching refers to the practice of tailoring instruction to meet the diverse needs, abilities, and learning styles of individual students within a classroom.	(73)	4
	Questioning	Questioning engages students, promotes critical thinking, and deepens understanding. By using open-ended and well-timed questions, teachers encourage active participation, assess comprehension, and stimulates higher-order thinking and problem-solving skills.	(74)	4
	Worked examples	Worked examples provide step-by-step demonstrations of problem-solving processes, helping students understand complex tasks. By observing correct methods and rationales, students can focus on learning key principles before attempting independent problem-solving.	(75, 76)	4
	Feedback	Providing timely, constructive feedback enables students to understand their progress and areas for improvement, enhancing learning outcomes.	(50, 65)	4
Teaching standard	Know students and how they learn	Understand the diverse needs, backgrounds, and learning styles of students to adapt teaching strategies effectively.	(50)	4
	Know the content and how to teach it	Master the curriculum content and employ appropriate teaching methods for student understanding.	(50)	4
	Plan for and implement effective teaching	Design well-structured lessons and implement teaching strategies that engage students and promote learning.	(50)	4
	Create and maintain supportive and safe learning environments	Foster a positive, inclusive, and safe classroom environment that encourages participation and student well-being.	(50)	4
	Assess, provide feedback and report on student learning	Use various assessment methods to monitor progress, provide constructive feedback, and report results clearly to students and stakeholders.	(50)	4

To support consistent data extraction, the research team compiled a reference list of terms and activities associated with physically active teaching and learning. This list included pedagogical approaches and tasks aligned with active teaching such as *experiential learning*, *role plays*, *hands-on learning*, *station-based activities*, *kinaesthetic learning*, *simulations*, *embodied learning*, *gallery walks*, and *scavenger hunts*. A full list of identified terms is provided in Supplementary Table 1.

#### 2.5.1 Lesson plans

A total of 145 lesson plan sequences were downloaded from the PebblePad platform. All submissions were de-identified, and each pre-service teacher and corresponding sequence of three lesson plans were assigned a unique identifier (e.g., *ID*1*_LP*1, *ID*1*_LP*2, *ID*1*_LP*3). All lesson plans were saved in a secure, password-protected Dropbox folder to ensure confidentiality and data security.

A macro-enabled Microsoft Excel workbook was developed to facilitate systematic data extraction. The first six columns of the workbook captured descriptive information, including the unique ID, file name, data extractor name, school year level (via dropdown menu), subject area (via dropdown menu), and notes for other subjects not listed. The next column recorded a binary response (Yes/No) to indicate whether any active teaching strategy was present in the lesson plan.

For lesson plans that included active strategies, additional fields were populated aligned with the domains and items presented in Table 1 above. These next columns were used to document specific information about active breaks and active lessons, including: the name of the strategy, the type of strategy, a description of the strategy, relevant quotes/text from the lesson plan, the phase of the lesson in which the strategy occurred, the associated teaching practice, and the relevant Australian Professional Teaching Standard. If no active strategy was identified in a lesson plan (i.e., Column G marked “No”), data extraction for that particular plan was deemed complete, and no further analysis was conducted for that entry.

This process was repeated for each of the three lesson plans submitted by a pre-service teacher, resulting in three rows of lesson plan data per participant (e.g., *ID*1*_LP*1, *ID*1*_LP*2, *ID*1*_LP*3).

The pre-service teachers' lesson plan assignments were evenly distributed among the research team (approximately 25 participants per team member) for independent analysis. To ensure consistency and rigour, the lead researcher conducted reliability checks on 10% of the lesson plans coded by each team member. Any discrepancies were discussed collectively until consensus was reached. Regular calibration meetings were held throughout the coding process to refine interpretations and maintain consistent application of the coding framework. This collaborative and iterative approach, combined with transparent documentation of coding decisions, enhanced both the reliability and validity of the data extraction process.

#### 2.5.2 Videos

In addition to the lesson plans, video reflections submitted by each pre-service teacher were collected and stored as part of the dataset. Each video was assigned a unique identifier linked to the participant's ID (e.g., ID1_V) to maintain confidentiality and enable accurate tracking.

The video reflections were first viewed in their entirety to develop a general understanding of the content and to identify key pedagogical elements related to active teaching and learning. Subsequent viewings involved a more detailed review, during which specific segments were flagged where pre-service teachers explicitly identified, described or demonstrated active pedagogical strategies. Verbatim excerpts from these segments were transcribed and entered into a structured Excel workbook, with relevant information recorded in the columns dedicated to video data, and captured specific references to active breaks, active lessons, and active learning environments, respectively. An additional column was used to record direct quotes related to active pedagogies as open-text responses, enabling qualitative analysis of pre-service teachers' understanding and application of active teaching approaches.

To enhance the trustworthiness of the qualitative analysis, the lead researcher co-coded 10% of the video reflections to ensure consistency in interpretation and application of the coding framework. The research team engaged in iterative viewing, peer debriefing, and reflexive discussions throughout the data extraction period to refine coding decisions and maintain analytical rigour.

### 2.6 Data analysis

#### 2.6.1 Lesson plans

Cross-checking of 10% of coded lesson plans indicated a high level of agreement among the research team (approximately 85%–90%), consistent with substantial inter-rater reliability (Cohen's *κ* ≈ 0.80). Discrepancies were minor and resolved through discussion to ensure consistency across all coded data.

The extracted lesson plan data, initially compiled in a Microsoft Excel workbook, was imported into Stata SE (version 18) (57) for analysis. The dataset was reshaped from a long to a wide format to facilitate variable creation and summary statistics. A descriptive analysis was then undertaken to quantify key features of the lesson plans submitted by the pre-service teachers. New variables were generated to calculate the total number of active breaks and active lessons included across the submissions. Frequency distributions were also produced to examine the prevalence of specific instructional strategies, the phase of the lesson in which these strategies were applied (e.g., introduction, body, or conclusion), and the types of teaching practices used, and teaching standards addressed.

#### 2.6.2 Videos

A descriptive thematic analysis (58) was conducted to examine the presence and characteristics of active pedagogical strategies within the pre-service teachers' video reflections. This analytical approach was chosen for its capacity to identify, organise, and describe patterns within qualitative data without applying interpretive frameworks beyond what was explicitly present in the content.

Data were initially extracted from the Excel workbook and transferred into a Microsoft Word document, which was subsequently imported into NVivo version 14 (59). An inductive coding process was employed, allowing for the identification of meaningful segments of text. Codes remained close to the participants' original language and captured explicitly stated or clearly observable pedagogical choices (e.g., “group work,” “scaffolding,” “experiential learning”). This open coding approach ensured that the analysis remained grounded in the data and reflected the participants' own perspectives.

Following initial coding, codes were reviewed and grouped into conceptually related categories, enabling the identification of broader patterns across the dataset. Each theme was carefully defined and refined to ensure conceptual clarity and to accurately represent the data (see Supplementary Table 2). To provide a descriptive overview, the frequency of occurrence for each theme was calculated using NVivo. Additionally, illustrative excerpts from the video reflections were selected to exemplify each theme, enhancing the richness and interpretability of the findings.

Credibility was supported through analyst triangulation, where two researchers reviewed and compared interpretations, and through the inclusion of verbatim excerpts to ensure authenticity and transparency in representing participants' perspectives.

## 3 Results

### 3.1 Lesson plans

**Figure 1:**
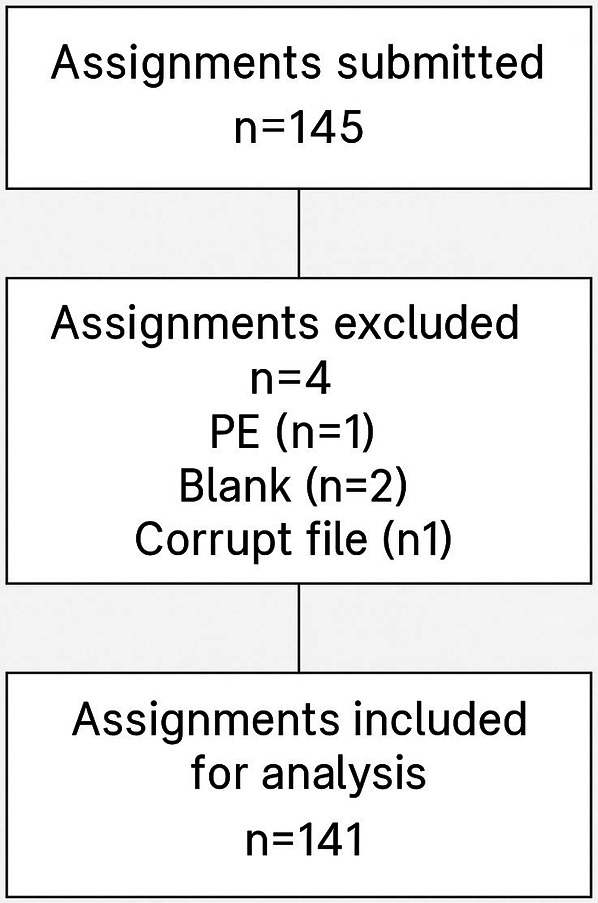
Assignment flow.

A total of 145 pre-service teachers submitted assignments for assessment. Four submissions were excluded due to incomplete or missing data, resulting in 141 assignments included in the final analysis (see Figure 1).

The pre-service teachers selected a variety of school year levels and curriculum areas for their lesson sequence assignments, with Grade 1 (19%) and English (35%) emerging as the most frequently selected options for year level and subject, respectively (see Table 2).

**Table 2:** Year levels and subjects of included lesson plans^a^.

Grade and subject	Frequency	Percent
Kinder	6	4.26
Prep	12	8.51
Grade 1	27	19.15
Grade 2	18	12.77
Grade 3	22	15.60
Grade 4	24	17.02
Grade 5	14	9.93
Grade 6	16	11.35
Kinder/Prep	1	0.71
Grade 3/4	1	0.71
TOTAL	141	100
English	50	35.46
Mathematics	26	18.44
Science	33	23.40
Humanities and social sciences	21	14.89
Languages	1	0.71
The Arts	4	2.84
Other	6	4.26
TOTAL	141	100

a Based on data extracted from first lesson plan only. All three lesson plans were intended to be for the same year level and subject, but some students (n = 13) did not follow this instruction.

Of the 141 PSTs, 125 (89%) incorporated at least one PAL strategy within their lesson plan sequences. Across all submissions, a total of 447 active strategies were identified, yielding an average of 3.17 active strategies per pre-service teacher, with more active breaks presented than active lessons (Table 3).

**Table 3:** Total and average active strategies per pre-service teacher, and the frequency of response for type, phase, teaching practices and teaching standards.

Domain	Active breaks	Active Lessons	Total
Total of active strategies included in lesson plans	312	135	447
Average number of active strategies included in lesson plans per pre-service teacher	2.21 (0.18)	0.96 (0.10)	3.17 (0.21)
Type^a^			
Energise	7		
Learn	6		
Manage	3		
Structure	123		
Transition	174		
Embodied learning		23	
Experiential learning		68	
Structural or incidental		44	
Phase^b^			
Introduction	39	10	49
Body	174	100	274
Conclusion	87	13	100
Teaching practices^c^			
Goal setting	0	0	0
Structuring lessons	236	64	300
Collaborative learning	47	40	87
Metacognitive strategies	0	18	18
Explicit teaching	1	0	1
Multiple exposures	6	25	31
Differentiation	1	0	1
Questioning	5	1	6
Worked examples	1	1	2
Feedback	22	2	24
Teaching Standards^c^			
Know students and how they learn	13	7	20
Know the content and how to teach it	18	49	67
Plan for and implement effective teaching	220	78	298
Create and maintain supportive and safe learning environments	9	1	10
Assess, provide feedback and report on student learning	52	14	66

a One Active Break was categorised as two types and therefore the total count here is increased by one compared to the total number of Active Breaks presented in row one above.

b This variable was not extracted for some of the active strategies (Active Breaks n = 12; Active Lessons n = 12) and therefore the totals here do not match those presented in row one above.

c These variables are multi-response and therefore the totals here will not match those presented in row one above.

Among the PAL strategies identified, active breaks were most commonly categorised as *transition-based* (*n* = 174), followed by *structure-based* strategies (*n* = 123). In the category of active lessons, *experiential learning* was the most frequently observed type (*n* = 68). The majority of active strategies were implemented during the *body* of the lesson, with 174 active breaks and 100 active lessons occurring within this phase.

In terms of associated teaching practices, *structuring lessons* was the most identified pedagogical purpose for both active breaks (*n* = 236) and active lessons (*n* = 64). With respect to alignment with the Australian Professional Standards for Teachers—Graduate Level, the standard most frequently addressed by both active breaks (*n* = 220) and active lessons (*n* = 78) was *Standard* 3*: Plan for and implement effective teaching and learning* (see Table 3).

### 3.2 Videos

Of the 141 pre-service teachers, approximately 50% (*n* = 71) explicitly referenced PAL in their in their video reflections in response to audience-specific prompts, for example, when explaining their teaching choices to a hypothetical colleague.

Through descriptive thematic analysis, references to PAL strategies were organized into eight overarching pedagogical themes (Table 4).

**Table 4:** Pedagogical Themes.

Pedagogical theme	Example code	References	Example quotes
Assessment	Formative assessment Active questioning Peer feedback	(4)	*Active approaches are great for formative assessment particularly for peer check ins discussions and peer feedback.*
			*Active questioning allows for an immediate understanding of where your students are in their learning.*
Collaborative learning	Collaborative learning Group work Teamwork and communication Peer interaction Cooperative learning	(23)	*Activity is great for transitioning students into cooperative learning.*
			*The use of activity for small group work activities works well to improve engagement.*
Constructivist approach	Constructivist Inquiry-based learning Play-based learning Project-based learning	(24)	*Explore concepts through inquiry and experiments—helps with critical and creative thinking*
			*Constructivist approaches help to build their own understanding*
			*Inquiry based approaches leads to deeper learning*
Embodied and Experiential learning	Embodied learning Experiential learning Real-world engagement Role-playing Simulation Performance-based learning	(17)	*Students actively interact with recycling and sustainability to experience it and make it more meaningful to their daily lives*
			*Specific maths topics are best taught through tactile experience that help solidify understanding*
Inclusive approaches	Culturally sensitive Inclusive learning 8 ways aboriginal pedagogy Reflective practice	(6)	*Being active aligns to the* 8 *ways aboriginal pedagogy via its hands on and reflective approach*
Integrated or multimodal learning	Multiple intelligences Visual representation Mulit sensory learning stations Visual and textural alignment	(4)	*The use of visual and textual approaches helps build deeper learning*
Learning through doing	Hands-on learning Kinaesthetic learning Manipulation Tactile experience Movement-based activities	(37)	*Art is inherently tactile and hands on, so that is the best way to teach it*
			*Active pedagogies which focus on hands on activities and interactive experiences allow students to take charge of their learning*
			*Physically interact with materials to enhance retention and understanding of elements*
			*Specific maths topics are best taught through tactile experience that help solidify understanding*
Planning and structuring	Scaffolding Active Transitions Explicit teaching 5 Es approach	(5)	*The use of active strategies really helps student to engage and explore as part of the* 5*E’s Model*

The most frequently cited theme was Learning Through Doing (*n* *=* 37), which encompassed hands-on learning, tactile experiences, and movement-based activities. This was followed by Constructivist Learning (*n* *=* 24), which included approaches such as inquiry-based learning, play-based learning, and project-based learning. The theme of Collaborative and Cooperative Learning appeared in *n* *=* 23 reflections, with examples such as group work, peer interaction, and cooperative tasks. Embodied and Experiential Learning was referenced *n* *=* 17 times and included strategies such as role-playing, simulations, and real-world learning. Inclusive Approaches were cited in *n* *=* 6 cases, with reflections referencing culturally responsive practices, such as the 8 Ways Aboriginal Pedagogy, and reflective practices. Planning and Instruction appeared *n* *=* 5 times, with references to instructional models (e.g., the 5Es), scaffolding learning, and explicit teaching techniques. Integrated and Multi-Modal Learning was noted in *n* *=* 4 instances, highlighting the use of visual aids, multi-sensory stations, and strategies aligned with multiple intelligences. Finally, Assessment appeared in *n* *=* 4 reflections, featuring formative techniques, active questioning, and peer feedback.

## 4 Discussion

This study examined how first-year pre-service teachers integrate PAL strategies into their lesson planning and reflections, using a mixed-methods design informed by pragmatic, constructivist, and practice theory perspectives (51, 52). By analysing both lesson sequence plans and reflective video submissions, the research provides important insights into how *TransformUs Higher Ed* principles are operationalised within curriculum design and lesson planning, and the extent to which active pedagogies are embedded into pre-service teacher practice.

Overall, the findings indicate a strong uptake of active teaching strategies, with 89% of the 141 analysed submissions incorporating at least one active pedagogical element. The most commonly implemented strategies were transition-based active breaks and experiential learning activities (9, 47), which were predominantly positioned during the main body of lessons to structure learning and maintain student engagement. These findings suggest that pre-service teachers are increasingly recognising the value of embedding physical activity into lesson flow, not simply as an adjunct or classroom management tool, but as a deliberate component of instructional design. This aligns with recent research on strategies that enhance the effectiveness of physically active learning programs (8). Transition-based active breaks, for example, were used in ways that moved beyond traditional “brain breaks” towards more intentional, learning-focused transitions that serve both cognitive and behavioural purposes (9).

Experiential activities similarly reflected a shift from incidental activity toward more meaningful “learning by doing.” This aligns closely with Kolb's Experiential Learning Theory (60, 61), which conceptualises learning as a cyclical process comprising concrete experience, reflective observation, abstract conceptualisation, and active experimentation. The prominence of hands-on, kinaesthetic, collaborative, and inquiry-based approaches observed in the reflective videos underscores pre-service teachers' growing orientation toward student-centred pedagogies, in which learners actively construct understanding through purposeful engagement with content and peers (3).

The alignment of these PAL strategies with constructivist learning theory was also evident across both data sources. Constructivism posits that learners construct knowledge actively through experience, problem-solving, and the integration of new information with prior knowledge (53). This philosophical stance was reflected in participants' deliberate inclusion of problem-based tasks, scaffolding for critical thinking, and a focus on personal meaning-making within lessons. It appeared that the pre-service teachers assumed the role of facilitators in their lesson planning, creating environments where students were encouraged to explore, collaborate, and reflect, characteristics central to meaningful knowledge construction (5, 62).

Further, the analysis identified consistent alignment between PAL strategies and established evidence-based teaching practices. Active strategies were embedded within clear lesson structures, supporting the sequencing of teaching and learning activities to scaffold understanding and maintain engagement (63). Collaborative learning was commonly incorporated, encouraging student cooperation through meaningful group tasks that fostered shared responsibility and peer-to-peer learning (64). Many lesson plans integrated metacognitive strategies, enabling students to plan, monitor, and evaluate their own learning processes, a practice well-supported by robust evidence for its positive impact on student outcomes (2). Multiple exposures to key concepts were evident, providing learners with repeated, varied opportunities to engage with and consolidate new knowledge (44). Finally, targeted feedback strategies were included, supporting both formative and summative assessment practices designed to guide student learning and inform instructional adjustments (65).

Importantly, these pedagogical choices align with the Australian Professional Standards for Teachers (50), particularly Standard 3, which focuses on planning for and implementing effective teaching and learning. The explicit inclusion of PAL strategies in lesson plans aligned with APST focus areas, demonstrates pre-service teachers' emerging ability to align individual teaching practice with broader professional and sector expectations (3). The reflective videos further reinforced this alignment, providing evidence of deliberate pedagogical reasoning and purposeful integration of active strategies as a core element of effective teaching practice.

Notably, English and mathematics were two of the most selected subjects for lesson planning. These are traditionally high-stakes disciplines, often assigned extended learning blocks. They also traditionally correspond with high amounts of sedentary learning. The integration of active strategies within these subjects represents a meaningful paradigm shift, reflecting an important bridging of the evidence base on PAL and academic outcomes with the practical skill of lesson design (14, 28). This movement toward embedding activity within core academic subjects reflects an important evolution in pre-service teacher thinking, challenging traditional norms and potentially contributing to both academic and health-related benefits for students. This approach aligns with research on strategies that enhance the effectiveness and impact of PAL (8). Ultimately, this research highlights the critical role of PAL-focused teacher education in developing effective educators capable of advancing students' academic outcomes through evidence-based, active pedagogies.

### 4.1 Strengths and limitations

This study offers several strengths. The use of an authentic assessment task grounded in real-world lesson planning provides strong ecological validity, while the large sample size enhances the generalisability of findings. The development of the analytic coding framework, informed by pedagogical theory and teaching standards, ensured a consistent and theoretically grounded approach to data analysis. Moreover, the systematic coding process supported reliable categorisation of diverse active strategies across submissions.

However, several limitations warrant consideration. First, it remains unclear to what extent these planned active strategies will transfer into actual classroom practice during placements, where contextual factors and institutional norms may influence implementation (8). Second, while the assessment task provided a scaffold for demonstrating pedagogical planning, it may have inadvertently constrained authentic expression of pedagogical reasoning, potentially steering participants towards compliance with assignment expectations rather than genuine enactment of teaching beliefs. The overrepresentation of English and mathematics lessons, with underrepresentation of other subject areas such as LOTE and technology, may also limit the breadth of generalisability across curriculum areas. Finally, although few participants opted out, their absence may have introduced bias, and demographic data that would have been collected via consent were unavailable.

### 4.2 Future research

Future research should build on these findings by following pre-service teachers into their professional placements, examining the adoption and implementation of PAL strategies in authentic teaching contexts. Longitudinal research could further explore how early adoption of active pedagogies during pre-service training translates into sustained professional practice and impacts teacher effectiveness and student outcomes over time.

## 5 Conclusion

This study provides promising evidence that integrating PAL strategies early in pre-service teacher planning can enhance pedagogical competence. Incorporating PAL-focused interventions within ITE programs positions lesson planning as a key step in developing effective, evidence-based teaching practices. Practical recommendations include embedding PAL activities across multiple curriculum subjects, providing structured guidance and examples in lesson planning workshops, and offering opportunities for pre-service teachers to reflect on and adapt these strategies in classroom simulations or practicum placements.

## Data Availability

The raw data supporting the conclusions of this article will be made available by the authors, without undue reservation.
