# Supplementary material for: From theory to practice: examining the influence of physically active learning on curriculum design and pedagogical planning in initial teacher education
Source: Front Sports Act Living. 2025 Nov 26;7:1719341. doi: 10.3389/fspor.2025.1719341 (PMC12689541; doi:10.3389/fspor.2025.1719341)
Supplement: Supplementary file 1 [file Table1.docx]

Supplementary Table 1**.** Potential PAL strategy search terms

| **Term / Strategy** |
| --- |
| Physically Active Learning (PAL) |
| Physical activity |
| Movement |
| Movement-based learning |
| Active learning |
| Active environment |
| Active lesson |
| Active break |
| Learning by doing |
| Kinaesthetic learning |
| Embodied learning |
| Embody |
| Whole-body learning |
| Dynamic learning |
| Motor-enriched instruction |
| Full-body engagement |
| Energetic engagement |
| Motion-based education |
| Experiential learning |
| Hands-on learning |
| Role plays |
| Simulations |
| Scavenger hunts |
| Obstacle courses |
| Puzzle-solving |
| Gallery walks |
| Movement-based storytelling |
| Story dramatization |
| Gesture-based learning |
| Learning stations |
| Learning through art |
| Concept mapping with movement |
| Mindful walking |
| Movement journaling |
| Spatial learning |
| Collaborative learning |
| Peer teaching |
| Peer feedback sessions |
| Think-pair-share |
| Fishbowl discussions |
| Quick active debates |
| Standing Classroom debates |
| Walking discussions |
| Role reversal (students as teachers) |
| Socratic walks |
| Cross-curricular activities |
| Creative problem-solving |
| Problem-based learning (PBL) |
| Project-based learning |
| Service learning |
| Exploration activities |
| Reflective activities |
| Interactive storytelling |
| Interactive quizzes |
| Flipped classroom activities |
| Mindfulness activities |
| Yoga for learning |
| Stretching exercises |
| Brain breaks |
| Mindful movement |
| Somatic learning |
| Emotionally embodied learning |
| Body-mapping ideas |
| Mind-body learning |
| Flexible seating |
| Standing desks |
| Tactile learning |
| Sensory-friendly classroom |
| Transformational seating |
| Mobile classrooms |
| Modular classroom layouts |
| Go Noodle (or similar program) |
| Choice-based learning centers |
| Chalk talk with movement |
| Interactive anchor charts |
| Tactile puzzles or manipulatives |

# Supplementary Table 2. Thematic Categories of Active Teaching Approaches

| **Category** | **Term / Strategy** |
| --- | --- |
| Hands-On and Kinaesthetic Learning | Hands-on learning |
|  | Kinaesthetic learning |
|  | Learning through doing |
|  | Manipulation |
|  | Learning through practical movement |
|  | Hands-on practical nature of activities based outside |
|  | Art is inherently tactile and hands on |
|  | Physically interact with materials to enhance retention and understanding |
|  | Specific maths topics best taught through tactile experience |
|  | Hands-on learning allows students to problem-solve ideas |
|  | Hands-on learning and simulations |
|  | Hands-on group work |
|  | Hands-on learning through the movement game |
|  | Hands-on learning activities |
|  | Hands-on exploration |
|  | Hands-on rotations |
|  | Get students out of their seats and moving |
|  | Active pedagogies focused on hands-on activities |
|  | Students actively engaging with the experiment physically |
|  | Hands-on engaging activities |
|  | Hands-on and play-based learning |
|  | Hands-on activities |
|  | Play-based activities |
| Constructivist and Inquiry-Based Learning | Constructivist |
|  | Constructivist hands-on approach |
|  | Constructivist pedagogy |
|  | Constructive approach helps build their own understanding |
|  | Inquiry |
|  | Inquiry-based learning |
|  | Inquiry-based to explore real-world scenarios |
|  | Inquiry and hands-on experiences |
|  | Inquiry-based thinking |
|  | Inquiry-based, constructivist |
|  | Inquiry-based, cooperative, play-based learning |
|  | Project-based learning |
|  | Explore concepts through experiments |
|  | Build understanding through exploration |
|  | Inquiry-based, collaborative learning, and explicit teaching |
|  | Inquiry-based learning, collaborative learning |
|  | Inquiry-based learning, direct questioning/instruction, and scaffolding |
|  | Inquiry-based, constructivist, formative assessment |
|  | Inquiry-based learning, critical and creative thinking |
|  | Questioning and evaluating |
|  | 5E Model / 5 E’s approach |
| Collaborative and Cooperative Learning | Collaborative learning |
|  | Small group work |
|  | Group work |
|  | Collaborative group work |
|  | Peer interaction |
|  | Group activities |
|  | Collaborative work |
|  | Collaboration activities |
|  | Collaboration in groups |
|  | Working as a team |
|  | Teamwork and communication |
|  | Transition into cooperative learning |
|  | Flexible grouping |
|  | Interact productively |
|  | Group discussion and collaboration |
|  | Group work and peer feedback |
| Embodied or Experiential Learning | Active learning |
|  | Active pedagogies |
|  | Active student participation and deeper learning through exploration |
|  | Students actively interact with recycling and sustainability |
|  | Create active learning environments to promote participation |
|  | Experiential learning |
|  | Experiential and cognitive-based learning |
|  | Performance-based pedagogy – role playing |
|  | Outside walk to ensure all students’ needs are being met |
|  | Connect what they are learning to the real world |
| Culturally Responsive and Embodied Approaches | 8 Ways Aboriginal Pedagogy |
|  | Embodied learning |
|  | Hands-on reflective approach |
| Integrated and Multi-Modal Learning | Multiple intelligences – use kinaesthetic elements |
|  | Visual aids |
|  | Visual learning |
|  | Inclusive and comprehensive learning environment |
|  | Interactive tasks |
|  | Peer teaching and reflection |
|  | Reflective approach |
| Instructional and Assessment Strategies | Scaffolding |
|  | Direct questioning/instruction |
|  | Explicit teaching |
|  | Formative assessment (check-ins and peer feedback) |
|  | Whole class discussions |
|  | Discussion with open-ended |
